# Genomic and induction evidence for bacteriophage contributions to sargassum-bacteria symbioses

**DOI:** 10.1186/s40168-024-01860-7

**Published:** 2024-08-01

**Authors:** Alexandra K. Stiffler, Poppy J. Hesketh-Best, Natascha S. Varona, Ashley Zagame, Bailey A. Wallace, Brian E. Lapointe, Cynthia B. Silveira

**Affiliations:** 1 Department of Biology, University of Miami, Coral Gables, FL 33146 USA; 2 Department of Veterinary Population Medicine, University of Minnesota, Saint Paul, MN 55108 USA; 3 Harbor Branch Oceanographic Institute, Florida Atlantic University, Fort Pierce, FL 34946 USA; 4 Department of Marine Biology and Ecology, Rosenstiel School of Marine, Atmospheric, and Earth Science, University of Miami, Miami, FL 33149 USA

**Keywords:** Bacteriophage, Biofilm formation, Induction, Primary producer, Metagenomics, Metagenome-assembled genome

## Abstract

**Background:**

Symbioses between primary producers and bacteria are crucial for nutrient exchange that fosters host growth and niche adaptation. Yet, how viruses that infect bacteria (phages) influence these bacteria-eukaryote interactions is still largely unknown. Here, we investigate the role of viruses on the genomic diversity and functional adaptations of bacteria associated with pelagic sargassum. This brown alga has dramatically increased its distribution range in the Atlantic in the past decade and is predicted to continue expanding, imposing severe impacts on coastal ecosystems, economies, and human health.

**Results:**

We reconstructed 73 bacterial and 3963 viral metagenome-assembled genomes (bMAGs and vMAGs, respectively) from coastal *Sargassum natans* VIII and surrounding seawater. *S*. *natans* VIII bMAGs were enriched in prophages compared to seawater (28% and 0.02%, respectively). *Rhodobacterales* and *Synechococcus* bMAGs, abundant members of the *S*. *natans* VIII microbiome, were shared between the algae and seawater but were associated with distinct phages in each environment. Genes related to biofilm formation and quorum sensing were enriched in *S*. *natans* VIII phages, indicating their potential to influence algal association in their bacterial hosts. In-vitro assays with a bacterial community harvested from sargassum surface biofilms and depleted of free viruses demonstrated that these bacteria are protected from lytic infection by seawater viruses but contain intact and inducible prophages. These bacteria form thicker biofilms when growing on sargassum-supplemented seawater compared to seawater controls, and phage induction using mitomycin C was associated with a significant decrease in biofilm formation. The induced metagenomes were enriched in genomic sequences classified as temperate viruses compared to uninduced controls.

**Conclusions:**

Our data shows that prophages contribute to the flexible genomes of *S*. *natans* VIII-associated bacteria. These prophages encode genes with symbiotic functions, and their induction decreases biofilm formation, an essential capacity for flexible symbioses between bacteria and the alga. These results indicate that prophage acquisition and induction contribute to genomic and functional diversification during sargassum*-*bacteria symbioses, with potential implications for algae growth.

Video Abstract

**Supplementary Information:**

The online version contains supplementary material available at 10.1186/s40168-024-01860-7.

## Introduction

Pelagic brown macroalgae of the genus *Sargassum* (class Phaeophyceae, common name sargassum) are distributed widely between the Gulf of Mexico, Loop Current, Gulf Stream, Sargasso Sea, Caribbean Sea, and tropical Atlantic Ocean [1, 2], where they provide substrate and refuge for a plethora of marine organisms [3]. The original species *Sargassum natans* (Linnaeus) and *Sargassum fluitans* (Børgesen) display distinct morphotypes, *S*. *natans* I, *S*. *natans* VIII, and *S*. *fluitans* III [4, 5], that harbor distinct microbial communities, respond differently to extreme environmental conditions, display distinct growth rates, and have consistent polymorphisms in certain genes [6–8]. However, marker gene analysis of these morphotypes compared with benthic sargassum in the Caribbean and Brazil indicates that the morphotypes belong to the same species, *S*. *natans* [9, 10]. During bloom seasons, sargassum can account for up to 18% of the particulate organic carbon in the upper water column, playing a significant role in the marine carbon cycle [11]. When sargassum reaches the coast at low rates, it brings nutrients to shore habitats, promoting dune development and acting as a natural fertilizer [12, 13]. However, sargassum biomass has increased dramatically since 2011 with the development of the Great Atlantic Sargassum Belt in the tropical Atlantic [1, 14, 15]. In June 2018, the Great Atlantic Sargassum Belt consisted of more than 20 million metric tons of sargassum biomass, which is projected to increase [1]. Compared to historical data, sargassum’s nitrogen content has increased by 35%, and phosphorus content decreased by 44%, suggesting that sargassum is phosphorus-limited and that terrestrial runoff promotes blooms [16]. The explosive sargassum growth has increased the frequency and severity of sargassum beaching events on the Atlantic coast of the USA, impacting coastal environment stability, tourism, local economies, and human health [17–25].

As with other primary producers, the microbiome of sargassum is likely necessary for its growth and productivity by facilitating nutrient uptake and recycling [26–28]. The sargassum microbiome is an open symbiotic system where the algal host tissue is constantly exposed to varying environmental conditions across its life history, allowing for the switching of symbiotic partners [29]. Sargassum microbiome composition changes as it moves from the open ocean to coastal areas, presumably reflecting the different oceanographic regimes and symbiotic needs [30]. Epiphytic bacteria associated with sargassum may provide the algae with fixed nitrogen and with phosphorus-derived methylphosphonate under phosphorus starvation conditions [31, 32]. In the oligotrophic Sargasso Sea, the sargassum microbiome is dominated by the orders *Rhodobacterales*, *Alteromonadales*, and *Flavobacteriales* [24, 30]. In the eutrophic coastal areas of Florida and the Caribbean, the genus *Vibrio* becomes a more prominent community member [30, 33]. These differences in community composition could indicate distinct roles of the microbiome in these two oceanographic contexts: *Rhodobacterales* and *Alteromonadales* commonly form mutualistic relationships that are necessary for the growth of several primary producers [34–37]. *Flavobacteriales* are also commonly associated with primary producers, where they convert high molecular weight macromolecules into low molecular weight molecules by breaking down algal exudates [34, 38]. On the other hand, *Vibrio* species isolated from sargassum have the genomic potential for pathogenicity, including genes related to adherence, chemotaxis, toxins, regulatory elements, nutritional resistance, etc. [25]. While these studies have shown that a complex microbiome is associated with sargassum, a functional genomic investigation of these microbial communities in the algae’s natural environment is lacking.

Viruses that infect bacteria (bacteriophages or phages for short) are best known for their lytic infections when they kill the bacterial host to release progeny. Yet, temperate phages are capable of lysogenic infection, where the phage integrates into the host’s genome, replicating as a prophage while the host cell divides. In host-associated microbial communities where bacterial densities and viral encounter rates are high, the lysogenic life cycle is prevalent [39]. Prophage acquisition can modify phenotypes and increase bacterial fitness through lateral gene transfer and changes in gene expression, a phenomenon called lysogenic conversion [40]. In animals, lysogenic conversion and bacteriophage-mediated gene transfer have been shown to facilitate colonization and cause the emergence of pathogenicity [41, 42]. Genes associated with lysogenic conversion are also widespread in marine microbial communities, where they may be involved in symbiotic functions by encoding genes for key metabolic pathways, antibiotic compounds, viral superinfection exclusion, and niche expansion [43–46]. The *Rhodobacterales* group that dominates the sargassum microbiome in oligotrophic waters, for example, displays a genomic architecture that suggests that mobile genetic elements and viruses significantly contribute to genome diversification. *Rhodobacterales* commonly exhibit the “swim-or-stick” phenotype, switching between a free-living lifestyle and association with a primary producer host [47]. Two key regulators of this switch are quorum sensing molecules [48–50] and genetic elements such as plasmids and genomic islands originating from viruses [51, 52]. These genetic elements encode genes related to biofilm formation, including the rhamnose operon [47, 51]. *Rhodobacterales* prophages associated with primary producer blooms encode genes of the rhamnose operon (*rfb*ABCD), which manipulates the sugar composition of the O-antigen of lipopolysaccharide [53–55]. Upon infection with different prophages, certain *Rhodobacterales* species exhibit enhanced host-associated growth [56]. Therefore, prophages can significantly impact symbiotic relationships within holobionts by conferring advantages to certain strains of bacteria, shaping relationships between bacteria and their eukaryotic hosts.

Here, we investigated the contribution of phages to the genomic diversity of coastal sargassum holobionts (*S*. *natans* VIII) compared to surrounding seawater bacterial communities using metagenomics. We hypothesized that prophages are more prevalent in sargassum and may contain genes involved in colonization and symbiosis between bacteria and algae. We also performed in-vitro experiments with an enriched sargassum microbiome to investigate the relationship between induction of prophages from sargassum bacteria and biofilm formation. Our study reveals how phages contribute to genome flexibility and symbiosis between bacteria and sargassum.

## Results

### Sargassum morphotype identification and bacterial community composition

**Fig. 1:**
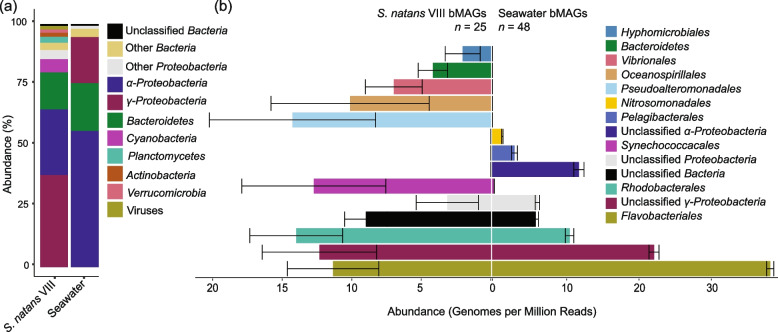
Bacterial community composition. Mean abundance of *S*. *natans* VIII and seawater bacteria at **a** the phylum level (PERMANOVA, *p* value = 0.01). **b** The abundance of bMAGs in genomes per million reads normalized by sample type. Bars display the mean abundances, and the error bars indicate the standard error (*S*. *natans* VIII *n* = 25 and seawater *n* = 48)

Sargassum microbiomes (< 8 µm dislodged from tissue, *n* = 5 algae strands), surrounding seawater bacteria (> 0.22 µm, *n* = 5 individual water samples), and surrounding seawater viruses (< 0.45 µm, *n* = 5 individual water samples) were collected nearshore on the South Florida coast near the city of Miami (Supplementary Data 1 for metagenomic sequencing and quality control data). Sargassum samples were identified as the morphotype *S*. *natans* VIII using an alignment-based approach. Briefly, complete mitochondrial genomes [57] and partial mitochondrial 16S genes [8] for *S*. *natans* I, *S*. *natans* VIII, and *S*. *fluitans* III were obtained from Genbank and aligned to co-assembled sargassum contigs from this study using BLASTn (see assembly steps in the “Methods” section). One contig matched the partial mitochondrial 16S gene of *S*. *natans* VIII with 100% identity, 100% coverage, and zero mismatches (Supplementary Data 2), confirming the identification of the samples used in our study as *S*. *natans* VIII. Herein, we refer to these algae by their common name, sargassum, as well as their specific morphotype, *S*. *natans* VIII. Sargassum and seawater displayed significantly different bacterial community compositions (PERMANOVA, *p* value = 0.01). The sargassum bacterial community was more diverse, rich, and even than the seawater bacterial community (Supplementary Table 1). *Alphaproteobacteria* was more abundant in seawater than in sargassum (56.18% and 29.34%, respectively; Welch’s T, *p* value = 0.00615; Fig. 1a; a complete list of bacterial phyla abundances is provided as Supplementary Data 3). *Gammaproteobacteria* composed 34.48% of the sargassum microbiome and 18.95% of the seawater microbiome (Fig. 1a). However, the difference in *Gammaproteobacteria* abundance between groups was not significant (Welch’s T, *p* value = 0.146) due to the variance of the bacterial abundances in the sargassum samples (high standard error, SE = 8.61%; Supplementary Data 3). Sargassum also harbored more *Cyanobacteria* (5.77%) than seawater (0.34%). At the order level, *Rhodobacterales*, *Alteromonadales*, *Vibrionales*, and *Flavobacteriales* composed 20.33%, 12.29%, 11.79%, and 10.79% of the sargassum microbial community, respectively (Supplementary Fig. 1). Conversely, seawater was dominated by *Rhodobacterales*, *Flavobacteriales*, *Cellvibrionales*, and *Pelagibacterales*, making up 44.49%, 12.11%, 6.76%, and 3.63% of the community respectively (Supplementary Fig. 1; a complete list of bacterial abundances at the order level is provided as Supplementary Data 4).

**Fig. 2:**
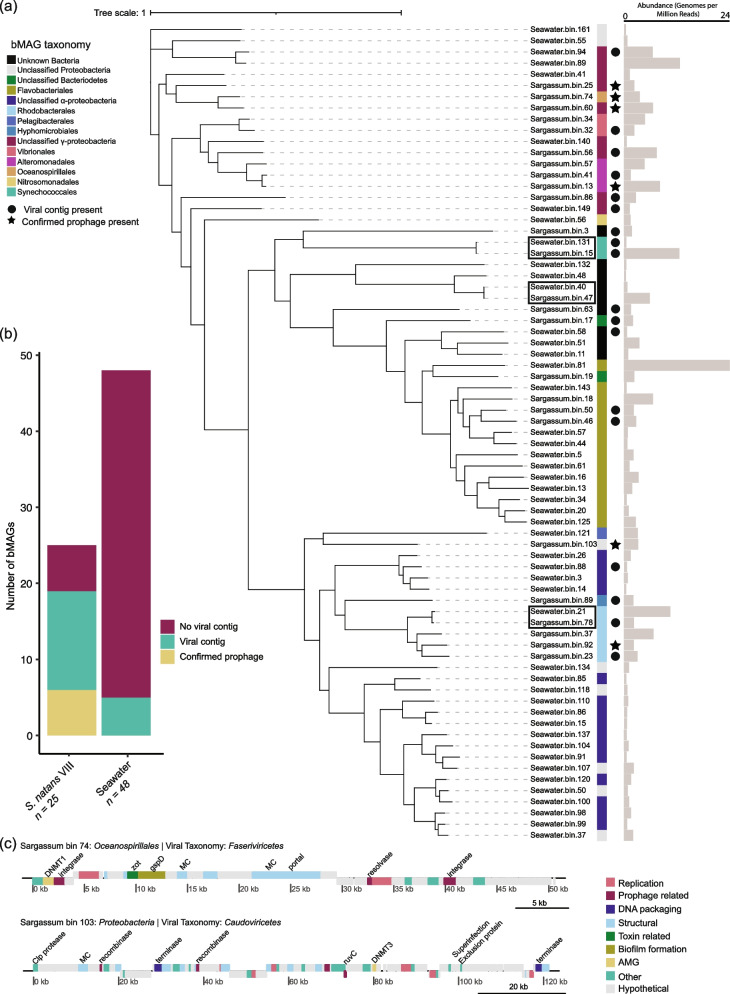
Prophage enrichment in *S*. *natans* VIII bacterial communities. **a** bMAG genome relatedness based on average nucleotide identity, with the presence of viral contigs within the bMAG represented by a circle and confirmed prophages indicated by a star. The black boxes indicate *S*. *natans* VIII and seawater bMAG pairs. The grey bars indicate bMAG abundance in genomes per million reads. **b** Frequency of bMAGs containing viral contigs and confirmed prophages in *S*. *natans* VIII (*n* = 25) and seawater (*n* = 48). **c** Genome architecture of two confirmed prophages from *S*. *natans* VIII selected based on gene content

Assembly and binning recovered 25 and 48 bacterial metagenome-assembled genomes (bMAGs, dereplicated at 95% average nucleotide identity) from sargassum and seawater, respectively (bMAG quality, taxonomy, abundance, and prophage information are available in Supplementary Data 5). Sixty percent of sargassum bMAGs were classified at the order level: four *Rhodobacterales*, three *Alteromonadales*, three *Flavobacteriales*, two *Vibrionales*, one *Hyphomicrobiales*, one *Oceanospirillales*, and one *Synechococcales*. In seawater, 31.25% were classified to the same level: 11 *Flavobacteriales*, one *Rhodobacterales*, one *Nitrosomonadales*, one *Pelagibacterales*, and one *Synechococcales*. The recovery of bMAGs in both sample types was comparable to the community structure determined by read recruitment (Fig. 1b). The most abundant bMAGs in sargassum were *Synechococcus* sp. WH 7803 (Sargassum.bin.15), *Pseudoalteromonas* sp. (Sargassum.bin.13), and an unknown *Gammaproteobacteria* (Sargassum.bin.56) with an average of 12.72, 8.21, and 7.46 genomes per million reads, respectively (Fig. 2a). Comparatively, the most abundant bMAGs in seawater were a *Flavobacteriaceae* (seawater.bin.81), an unknown *Gammaproteobacteria* (seawater.bin.89), and a *Rhodobacterales* (seawater.bin.21) with 24.43, 12.85, and 10.65 genomes per million reads, respectively (Fig. 2a).

### Prophage enrichment in sargassum bacterial communities

The 73 dereplicated bMAGs were clustered based on their average nucleotide identity and screened for potential prophages using VIBRANT. Three bMAGs were shared between sargassum and seawater: one *Synechococcales*, one *Rhodobacterales*, and one unclassified bacterium (Fig. 2a). Among the 25 bMAGs from sargassum, 80% contained viral genome fragments compared to only 12.5% of the 48 seawater bMAGs (Fig. 2b). To confirm that these bMAGs contained prophages, the contigs where viruses were identified were screened for bacterial flanking regions using VIBRANT and CheckV. Seven sargassum bMAGs encoded prophages, compared to only one in seawater bMAGs. Upon further analysis, two prophages were likely gene transfer agents, one in sargassum and one in seawater. Therefore, zero seawater and six sargassum bMAGs (24%) encoded confirmed prophages (Fig. 2b). The higher frequency of prophage identification in sargassum was not a byproduct of bMAG completion, as the difference between sargassum (mean = 81%) and seawater (mean = 77%) bMAG completion was not significant (Two-sample *T* test, *p* value = 0.343; Supplementary Fig. 2).

Among the bMAGs shared between sargassum and seawater, a sargassum *Rhodobacterales* bMAG contained viral genome fragments (genomic sequences identified as viral and binned with bacterial MAGs), while the seawater counterpart did not (Fig. 2a, bins outlined in black). A seawater *Synechococcales* bMAG contained one viral genome fragment, compared to two in the sargassum, one that matched its seawater counterpart and one that was exclusive. This *Synechococcales* bMAG comprised 0.28% of the community in seawater and 12.72% in sargassum. However, we could not confirm the bacterial flanking regions for these viral genome fragments. The six sargassum bMAGs that contained confirmed prophages included one *Rhodobacterales*, one *Oceanospirillales*, one unclassified *Proteobacteria*, two unclassified *Gammaproteobacteria*, and one *Pseudoalteromonas* sp. (Fig. 2a). Notably, the second most abundant bMAG in the sargassum samples, the *Pseudoalteromonas* sp., contained two unique prophages. The first and third most abundant bMAGs (*Synechococcus* sp. WH 7803 and an unclassified *Gammaproteobacteria*) contained viral genome fragments, but bacterial flanking regions could not be confirmed. Prophages in sargassum bMAGs encoded several genes that could manipulate their host’s metabolism and ecology (Fig. 2c). These include superinfection exclusion genes, DNA methyltransferase 1 (*DNMT1*), DNA methyltransferase 3 (*DNMT3*), and methylenetetrahydrofolate dehydrogenase (*folD*). These prophages also encoded genes related to biofilm formation and O-antigen biosynthesis such as general secretion pathway protein D (*gsp*), carbon storage regulator A (*csrA*), and other genes that can contribute to host virulence such as *zot* and FIC proteins (Fig. 2c).

### Viral community composition and infection strategies

**Fig. 3:**
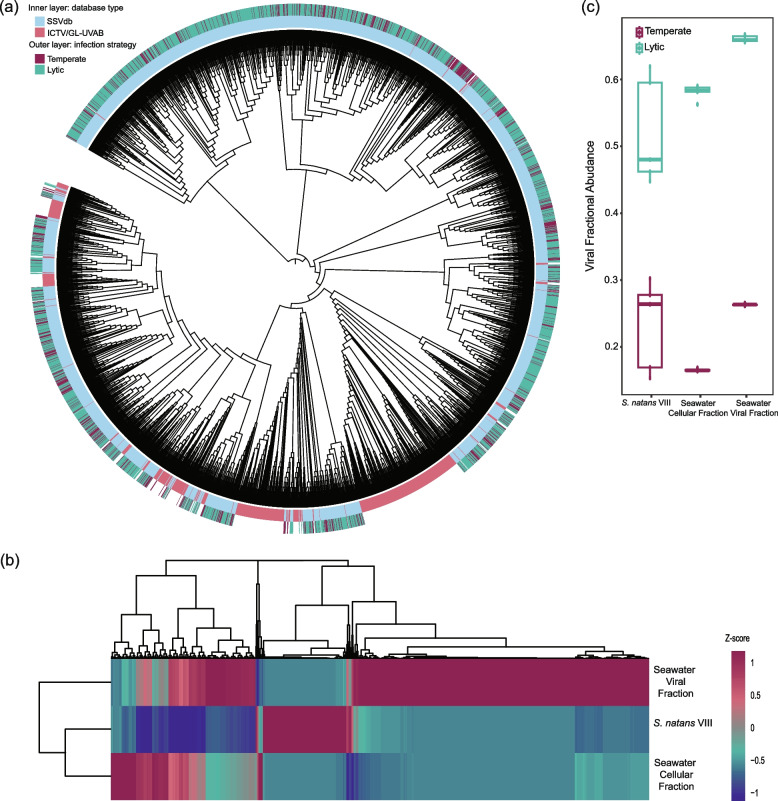
Viral community composition and infection strategies. **a** The viral proteomic tree was generated using an all-*versus*-all protein comparison and DICE distance using the viruses in the *S*. *natans* VIII and seawater database (SSVdb, light blue, this study) and reference viruses from the ICTV and GL-UVAB (pink) databases (databases indicated in the inner ring). **b** Viral fractional abundances in *S*. *natans* VIII, seawater cellular fraction, and seawater viral fractions (median values normalized by sample type using a z-score function, where magenta corresponds to highly abundant viruses and dark blue corresponds to rare viruses). **c** Abundances of temperate (magenta) or lytic (teal) viral fractional abundances per sample type (boxes display the median, first and third quartiles, and minimum and maximum values excluding outliers) (Lytic viruses: Welch’s T, *p* values = 1.098e-05 and 0.01734, for seawater cellular fraction and *S*. *natans* VIII, respectively) (Temperate viruses: Welch’s T, *p* value = 0.08697)

In addition to bMAG viruses, we identified 31,982 putative viral genomes across all contigs from the *S*. *natans* VIII, seawater cellular fraction, and seawater viral fraction dereplicated at 98% average nucleotide identity (Number of viruses and infection strategy per sample type are shown in Supplementary Table 2). Viral contigs were binned into viral metagenome-assembled genomes (vMAGs) within each sample type and all vMAGs and unbinned contigs longer than 10 Kbp or classified as medium or high quality based on their genome annotations were dereplicated at 95% average nucleotide identity and combined into the sargassum and seawater viral database (SSVdb) (Fractional abundances and infection strategy for each of the SSVdb viruses are provided in Supplementary Data 6). The SSVdb was composed of 3963 unique viral genomes belonging to three viral realms, *Duplodnaviria* (*n* = 2838), *Monodnaviria* (*n* = 1), and *Varidnaviria* (*n* = 42) (Fig. 3a), of which only 7% were related to ICTV viruses (minimum of 50% average amino acid identity and 70% shared protein-encoding genes). The seawater viral community had the highest diversity, richness, and evenness compared to sargassum and the seawater cellular fraction (Supplementary Table 3). The dominant and rare viruses in these three different environments are distinct from one another (Fig. 3b). The three most abundant viruses from sargassum were predicted to infect *Pseudoalteromonas*, *Flavobacteriales*, and an unclassified *Alphaproteobacteria* (host prediction shown in Supplementary Table 4). One of these viruses was identified as temperate based on the presence of an integrase gene. The three most abundant viruses identified in the seawater cellular fraction were predicted to infect a *Marinomonas* sp. and unknown bacterial bMAGs, and the three most abundant viruses from the seawater viral fraction were predicted to infect a *Rhodobacterales*, an unknown *Alphaproteobacteria*, and an *Alteromonas* sp. Lytic viruses were more abundant in the seawater viral fraction (mean = 0.661, *SD* = 0.00482) compared to the seawater cellular fraction (Welch’s T, *p* value = 1.098e-05, mean = 0.581, *SD* = 0.0108) and *S*. *natans* VIII (Welch’s T, *p* value = 0.01734, mean = 0.521, *SD* = 0.0801), which had a greater proportion of temperate viruses (Fig. 3c). However, the fractional abundance of temperate viruses was not different between *S*. *natans* VIII (mean = 0.234, *SD* = 0.0672) and the seawater cellular fraction (mean = 0.166, *SD* = 0.00185; Welch’s T, *p* value = 0.08697) due to the greater variance of the *S*. *natans* VIII viral fractional abundances (high standard deviation in Fig. 3c).

### Viral-encoded metabolic and biofilm-related genes

The SSVdb viruses encoded 1108 metabolic genes in 55 KEGG pathways (genes belonging to metabolic pathways and the phages encoding these genes can be found in Supplementary Data 7). Genes in five pathways were unique to *S*. *natans* VIII viruses and absent from seawater: beta-alanine metabolism, pantothenate/CoA biosynthesis, lipoarabinomannan biosynthesis, carbon fixation in prokaryotes, and benzoate degradation. There were no genes in unique pathways in seawater. The seawater cellular fraction displayed genes in 18 metabolic pathways at higher abundance, in contrast to 17 pathways, including those unique, in sargassum (Supplementary Fig. 3). Interestingly, 116 phages in the SSVdb encoded the gene for the phosphate starvation-induced protein PhoH and several others encoded proteins PhoR, PhoD, and PstS. PhoR is a phosphate regulon sensor histidine kinase, PhoD is an alkaline phosphatase, and PstS is a part of a phosphate-specific transport system. Further, we also identified one virus that encoded ArsR, the trans-acting regulatory protein of the arsenic resistance operon *ars*.

**Fig. 4:**
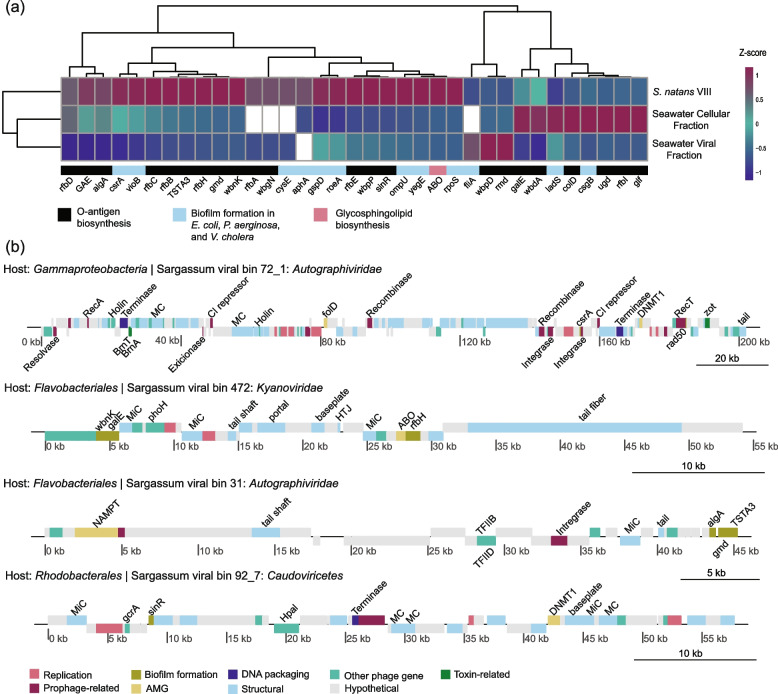
*S*. *natans* VIII-associated viruses encoding biofilm-related genes. **a** Abundance of viruses encoding genes involved in O-antigen biosynthesis (light blue), glycosphingolipid biosynthesis (pink), or biofilm formation pathways (black). The heatmap was normalized by gene using a z-score function. Abundances are displayed as the median (*n* = 5 for each sample type) of the sum of the fractional abundances of all viruses encoding that gene in a metagenome. **b** Genome plots of four *S*. *natans* VIII-associated viruses that encode genes related to O-antigen synthesis and biofilm formation

Within the SSVdb, 36 genes related to O-antigen biosynthesis, glycosphingolipid biosynthesis, and other biofilm formation pathways were identified in 172 viruses (Fig. 4a; phages and their biofilm genes are available in Supplementary Data 8). Forty-three viruses encoded more than one biofilm formation gene, with one having five, and 69.44% of these genes were encoded by viruses in *S*. *natans* VIII. When comparing the sargassum and seawater cellular fraction, 19 viruses with biofilm-related genes were unique to *S*. *natans* VIII compared to only 10 in the seawater viral fraction and none in the seawater cellular fraction. One gene, *mucR*, encodes a diguanylate cyclase involved in the regulation of alginate production during mucoid (biofilm-forming) growth of *P*. *aeruginosa* is not depicted in Fig. 4a because it was unique to two viruses found only in *S*. *natans* VIII, thus preventing the scaling and clustering of the data. Overexpression of this gene leads to auto-aggregation and the formation of highly structured biofilms accompanied by the suppression of flagellum-based motility [58]. The glycosyltransferase *ABO* was encoded by the most abundant virus encoding biofilm genes in *S*. *natans* VIII (Fig. 4b). Other abundant biofilm genes, encoded either by the most abundant viruses or multiple viruses in *S*. *natans* VIII, are the PadR family transcriptional regulator (*aphA*), O-antigen biosynthesis alpha-1,2-fucosyltransferase (*wbnK*), CDP-4-dehydro-6-deoxyglucose reductase (*rfbH*), and GDP-l-fucose synthase (*TSTA*3). Four quorum sensing-related genes were present in the SSVdb viruses. The acyl homoserine lactone synthase *raiI* and the quorum-sensing system regulator *bjaR1* were unique to viruses associated with *S*. *natans* VIII.

### Sargassum microbiome prophage induction and biofilm formation

To experimentally test the biofilm formation capabilities of the sargassum bacterial community and the potential viral roles indicated by the genomic analyses, we generated a sargassum bacterial enrichment for in vitro experiments. First, we collected additional sargassum samples (150 g, including all three morphotypes identified using the morphological classifications from Parr 1939), from the same location and scraped the algal tissue with 0.02 µm-filtered and autoclaved artificial seawater to dislodge tightly associated microorganisms. The scraped material was filtered through a 100 µm mesh and double-washed by centrifugation. Epifluorescence microscopy showed that this sargassum bacterial enrichment contained 1.4 × 10^6^ bacterial cells/mL and that viral-like particles (VLPs) were not detectable (Supplementary Fig. 4).

**Fig. 5:**
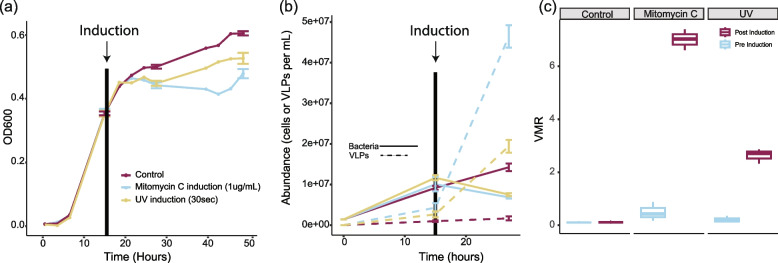
Planktonic prophage induction in sargassum-associated bacteria. **a** Growth and induction of bacterial enrichments obtained from sargassum. The vertical solid black line indicates the induction event. Standard error bars indicate the time points where samples were taken for microscopy (*t* = 15 h and *t* = 27 h) and the final growth reading at 48 h. (Control *n* = 5, UV *n* = 5, mitomycin C *n* = 5). **b** The abundance of bacterial cells (solid line) and viral-like particles (dashed line) at the beginning of the experiment, before induction (*t* = 15 h), and 12 h post-induction (*t* = 27 h) (ANOVA, *p* value = 4.01e-04 and 2.18e-08 for differences in cells and VLP abundances, respectively). Standard error bars are present before the induction event (*t* = 15 h) and the final time point (*t* = 48 h) (Control *n* = 3, UV *n* = 3, mitomycin C *n* = 3). **c** Virus-to-microbe ratio (VMR) before induction (teal) and post-induction (magenta) for each treatment group (ANOVA, *p* value = 1.36e-10; Tukey’s HSD, *p* value = 4.3e-12, for mitomycin C)

We conducted an induction assay with the sargassum bacterial enrichment to test if the prophages identified in sargassum-associated bacteria are intact and inducible. Exposure of the sargassum bacterial community to mitomycin C and UV radiation led to successful induction of bacteria in planktonic growth, as indicated by a drop in optical density at 600 nm (Fig. 5a). Epifluorescence microscopy corroborated these results showing that induced samples had a decrease in bacterial cell counts and an increase in viral-like-particles (cell abundance decreased by 1.55× and 1.47× and the viral abundance increased by 7× and 11× in the UV and mitomycin C groups, respectively; ANOVA, *p* value = 4.01e-04 and 2.18e-08 for differences in cells and VLP abundances, respectively, Fig. 5b). This led to an increase in the virus-to-microbe-ratio, indicating an induction event (Fig. 5c). Mitomycin C was a stronger inducer than UV exposure (Fig. 5c). All pre-induction groups did not differ in virus-to-microbe ratios (Tukey’s HSD, *p* values = 0.758 for UV versus mitomycin C, *p* value = 0.994 for UV versus control, and *p* value = 0.462 for mitomycin C versus control).

**Fig. 6:**
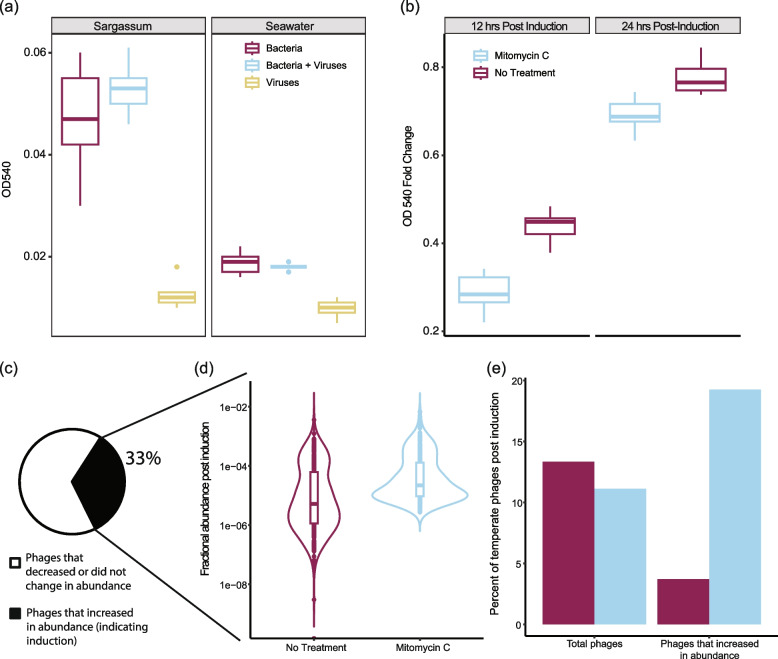
Biofilm prophage induction and resistance to external viral predation of sargassum bacteria. **a** Biofilm growth of the sargassum bacterial enrichment when grown in 10% sargassum-supplemented seawater or seawater (ANOVA, *p* value = 3.02e-06; Tukey’s HSD, *p* values = 5e-07) with and without the addition of free viruses concentrated from seawater (Tukey’s HSD, *p* value = 0.513) (all groups *n* = 5). **b** Fold change in biofilm formation by the sargassum bacterial enrichment when grown in 10% sargassum-supplemented seawater induced with mitomycin C (light blue) or not induced (magenta) (Control *n* = 5, mitomycin C *n* = 5) (ANOVA, *p* value = 1.69e-05; Tukey’s HSD, *p* value = 2.495e-04 for 12 h post-induction). **c** Percent of viruses that increased or decreased/did not experience a change post-induction when comparing mitomycin C and the control samples. **d** Fractional abundances for the 33% of viruses that increased post-induction, in mitomycin C (light blue) and the control samples (magenta) (Wilcoxon rank-sum *p* value = 2.2e-16). **e** Abundance of temperate phages post-induction in the control (magenta) and mitomycin C (light blue) between the total phages identified and the phages that increased with mitomycin C addition

We tested the sargassum bacterial community’s capacity for biofilm formation in two biofilm assays. For the first assay, the community was grown in either autoclaved seawater, sargassum-supplemented seawater, or autoclaved seawater with a viral concentrate obtained from the water surrounding sargassum mats (filtered through a 0.45 µm filter and concentrated using a 100 kDa tangential filter). Biofilm formation varied between the different conditions (Fig. 6a; ANOVA, *p* value = 3.02e-06). The bacterial community increased biofilm production by 2.5× when grown in the presence of sargassum-supplemented seawater (Tukey’s HSD, *p* value = 5e-07). The addition of external seawater viruses did not impact biofilm formation (Tukey’s HSD, *p* value = 0.513). For the second assay, the community was grown in sargassum-supplemented seawater, and after 12 h of growth, the prophages were either induced with 1 µg/mL of mitomycin C or received no treatment. Biofilm formation differed between the induced and non-induced samples (ANOVA, *p* value = 1.69e-05). Twelve hours post-induction, the bacterial community decreased biofilm formation by 1.5× compared to the non-induced treatment (Tukey’s HSD, *p* value = 2.495e-04; Fig. 6b). VLPs were detected in the induced samples (Supplementary Fig. 5).

Prophage induction in the second biofilm assay was verified by metagenomic sequencing of control and induced samples before and after induction (*n* = 5 for each treatment and time point). A total of 909 viral populations were identified in the assay, with 33% showing a significant increase in abundance after induction with mitomycin C compared to control (Fig. 6c). The abundance of these viruses increased by 4× on average in the induction treatment versus control (Wilcoxon rank-sum *p* value = 2.2e-16, Fig. 6d). Among these viruses, 19.25% were classified as lysogenic, compared to 3.71% in the control samples (Fig. 6e). Due to the limited dataset in this experiment resulting from low coverage sequencing (909 viral populations, mostly low quality, versus 3,963 medium and high-quality genome fragments in the SSVdb), only one biofilm gene was identified among them, *yhjH*, encoding a c-di-GMP phosphodiesterase involved in biofilm formation in *Escherichia coli*.

## Discussion

### Sargassum bacterial community

In the western tropical Atlantic and Caribbean Sea, where sargassum growth is exacerbated, the algae tissues have a higher N:C ratio compared to the Sargasso Sea, indicating that terrestrial nutrient enrichment contributes to sargassum overgrowth [59–61]. However, the ∂^15^N signatures in sargassum sampled on the East Coast of the USA were lower than predicted for a strong terrestrial signature. Nitrogen fixation by pelagic sargassum-associated *Cyanobacteria* is a possible explanation for the low ∂^15^N signal [31, 62, 63]. Our results support this hypothesis by showing that *S*. *natans* VIII sampled on the East Coast of the USA is enriched in *Synechococcus* spp. and other *Cyanobacteria*, some of which can fix atmospheric nitrogen (Fig. 1a and Supplementary Fig. 1). Previous observations showed that *Cyanobacteria* was a low-abundance member of sargassum microbiomes in the oligotrophic Sargasso Sea, increasing in abundance in the Caribbean [24, 33]. These bacteria may provide the sargassum host with fixed nitrogen while benefiting from heterotrophic growth on carbon released by the algae and utilizing other compounds released by heterotrophic bacteria in the holobiont [64, 65]. Future studies are necessary to quantify the nitrogen fixation and carbon metabolism in *Cyanobacteria* associated with coastal and open ocean sargassum.

The enrichment of *Vibrionales* in coastal *S*. *natans* VIII observed here was also observed in previous studies [25, 30]. *Vibrio* spp. are predicted to thrive in warm coastal waters as they favor higher temperatures and increased nutrient loads [66]. Therefore, an increase in sargassum growth may be coupled with exacerbated *Vibrio* growth under projected climate change-induced sea temperatures and hurricanes. Here, we identified potentially pathogenic *Vibrio* (Supplementary Fig. 6). However, whether sargassum stimulates the *Vibrio* spp. growth or if the high *Vibrio* spp. abundance is simply an effect of warmer and nutrient-rich coastal waters is unclear. Disentangling the cause and effect in this relationship is essential for securing coupled human-environmental health and will require field and laboratory studies with sargassum-associated *Vibrio* species.

### Prophage enrichment in sargassum bacteria

*S*. *natans* VIII bMAGs contained more viral genome fragments, with 20 out of 25 bins containing viral sequences, six of them (24%) being confirmed prophages (Fig. 2b), compared with none in seawater. This enrichment was observed despite the lower recovery of bMAGs from sargassum (*n* = 25), almost half of that from seawater. The difference in recovery is likely because a large portion of the reads in the *S*. *natans* VIII metagenomes belong to the alga, which complicates the bMAG binning process [67]. *S*. *natans* VIII metagenomes also had a slightly higher abundance of temperate viruses compared to seawater, although with high variability (large standard deviation in Fig. 3c). The variance in the *S*. *natans* VIII samples is likely due to heterogeneity of microbial biofilms on the algae surface, as observed in other holobionts [68]. The enrichment in prophages and temperate phages in sargassum is consistent with the prediction that bacterial communities associated with eukaryotic hosts are enriched in lysogens compared to free-living communities [69]. High lysogeny in host-associated microbiomes is likely to arise due to high encounter rates at high microbial densities [39]. Upon lysogenization, prophages can contribute to the flexible genetic repertoire of bacteria involved in open symbioses, where bacterial symbionts are swapped depending on environmental conditions and host needs [29]. This would be precisely the case of the *S. natans* VIII microbiome sampled here, which is dominated by extracellular symbionts exposed to the outside environment.

### Abundant sargassum phages encode genes for biofilm formation, quorum sensing, and phosphorus starvation response

Phages and other viruses infect many prokaryotic organisms that form biofilms worldwide [70]. A plethora of genes in multiple metabolic pathways encoded by *S*. *natans* VIII phages are shared with viruses in other marine biofilms [70]. For example, genes related to photosynthesis, purine metabolism, polyketide sugar biosynthesis, pantothenate/CoA biosynthesis, acarbose and validamycin biosynthesis, streptomycin biosynthesis, ubiquinone and other terpenoid-quinone biosynthesis were present in *S*. *natans* VIII biofilms and in other marine biofilms across oceans [70]. This may suggest that viruses associated with biofilms encode genes to help them exploit biofilm-forming prokaryotic organisms through multiple mechanisms. Additionally, genes involved in O-antigen nucleotide sugar biosynthesis are present in many marine biofilms [70] which was also seen in this study, where genes encoding enzymes in the O-antigen biosynthesis pathway necessary for biofilm formation were enriched in *S*. *natans* VIII phages (Fig. 4a). These data suggest that viruses enhance biofilm formation in their bacterial hosts and facilitate their association with *S*. *natans* VIII and with marine biofilms in general [70, 71]. Yet, some of these molecules, such as the O-antigen and the outer membrane proteins like OmpU, are also common phage recognition sites, and we cannot rule out the possibility that viruses carry these genes to manipulate host recognition sites to exclude additional phage infections [72]. The identification of phage-encoded quorum-sensing molecules further suggests that *S*. *natans* VIII phages modify the information relay of environmental stimuli that promote biofilm formation [73, 74]. Phage-encoded quorum sensing molecules could also allow for viral eavesdropping and communication of bacterial community composition, impacting the viral decision between lysis and lysogeny [75].

Here, we also observed phage-encoded genes involved in the phosphorus starvation response (*phoH*, *phoR*, and *phoD*). This suggests that not only epiphytic bacteria but potentially the phages that infect them may contribute to phosphorus uptake and cross-feeding between bacteria and their algal host by converting organic phosphorus compounds such as methylphosphonate to bioavailable inorganic phosphorus, providing *S*. *natans* VIII with a needed limiting nutrient [32]. Tightly coupled to the phosphorus starvation response is the arsenic resistance pathway, as As(V) is a chemical analog to phosphorus [76]. Due to this, sargassum bioaccumulates arsenic. In addition to genes related to phosphorus uptake, we also found one virus that encoded *arsR*, which regulates the arsenic resistance response in bacteria, suggesting that phages could also be bolstering bacterial responses to toxic metals, which has been seen in soil microbiomes [77].

### Biofilm formation and inducible prophage enrichment of the sargassum microbiome

Biofilm formation by sargassum-associated bacteria increased with the supplementation of seawater with sterile sargassum media, and was not altered upon the addition of free seawater viruses (Fig. 6a). These data suggests that the sargassum-derived bacterial community is largely resistant to lytic phage infection by exogenous seawater phages, potentially through superinfection exclusion conferred by integrated phages [72]. Prophages were inducible by UV exposure or mitomycin C treatment of sargassum bacteria grown planktonically (Fig. 5a–d). Remarkably, the biofilm formation of sargassum bacteria decreased upon prophage induction with mitomycin C (Fig. 6). Prophage induction in other systems has been shown to initiate bacterial biofilm decay [78]. This initial bacterial killing depletes the biofilm but, in some microbiomes, induction leads to long-term expansion of the biofilm by releasing nutrients via viral lysis [71]. This may have been the case in our experiment, as biofilm levels recovered to values close to the control after 24-h post-induction. Longer periods of biofilm growth post-induction will be needed to determine the role of phage induction on long-term biofilm progression. It cannot be ruled out that, in addition to prophage induction, mitomycin C may also have impacted biofilm formation through simple cell death without viral production. Prophages have been demonstrated to mediate the switch between a planktonic and a biofilm lifestyle in *Roseobacter*, a dominant bacterial group in sargassum [56]. The data described here suggests that this mechanism may be a key modulator of bacterial symbiosis with sargassum.

## Conclusion

Here, we demonstrate that the bacterial community recovered from coastal *S*. *natans* VIII is enriched with prophages and that many bacteriophages in this system, temperate and lytic, have the genetic potential to rewire interactions between the microbiome and the algae. In vitro evidence shows that prophages associated with sargassum are inducible, indicating that they are active members of the sargassum holobiont, and not “domesticated” genomic islands. Additionally, the bacterial biofilm producers of sargassum are not impacted by external viral predation but are impacted by prophage induction, suggesting that these prophages may have a direct role in biofilm formation and superinfection exclusion. The sargassum holobiont is an open symbiotic system, as it is constantly exposed to changing abiotic and biotic conditions with reshuffling of symbionts. Based on the data presented here, we propose that prophages modulate the flexible bacterial symbioses with sargassum, which may affect sargassum’s growth and niche expansion.

## Methods

### Sampling

Samples were collected in the coastal waters on April 14th, 2021, just before beaching (25°42′52.4′′N 80°09′01.0′′W). We collected five sargassum specimens for metagenomes, five 1 L seawater samples for metagenomes, and five 2 L seawater samples for viromes. Samples were placed into autoclaved glass collection jars and kept in a cooler until laboratory processing within 2 h of sample collection.

Sargassum specimens were rinsed thoroughly with DI water to remove the seawater microbiome and loosely associated bacteria, placed into 15 mL of 3.3% 0.02 µm-filtered artificial seawater, and vortexed for 20 min to dislodge the bacteria tightly associating with algal surfaces. This sargassum suspension was filtered through a 47 mm 8 µM Cyclopore track-etched membrane (Whatman, Milwaukee, USA), concentrated using a 100 kDa Amicon (Millipore, Burlington, USA) down to 500 µL, and stored at −20 °C until DNA extraction. DNA extractions of the sargassum microbiome were performed with a DNeasy Blood and Tissue Kit (QIAGEN, Germantown, USA). DNA concentrations were quantified using a Qubit 2.0 Fluorometer using a High-Sensitivity dsDNA kit (Thermo Fisher Scientific, Waltham, USA).

Five 1 L seawater samples were prefiltered through a 47 mm 8 µm Cyclopore track-etched membrane (Whatman, Milwaukee, USA) and collected on a 0.22 µm Sterivex filter (Millipore Sigma, Burlington, USA). The flow-through was discarded, and the 0.22 µm Sterivex filter with the bacterial community was frozen at −20 °C until DNA extraction. DNA extractions used a modified procedure using the Nucleospin Tissue Kit (Macherey-Nagel, Duren Germany). Briefly, T1 buffer and Proteinase K were added, and filters were incubated at 55 °C overnight, followed by adding B3 solution and another incubation at 70 °C for 30 min. Lysates were extracted from the filters with a 2 mL syringe, followed by the rest of the standard kit instructions. DNA concentrations were quantified using a Qubit 2.0 Fluorometer using a High-Sensitivity dsDNA kit (Thermo Fisher Scientific, Waltham, USA). Hereafter, these samples are referred to as seawater cellular fraction.

Five 2 L seawater samples were prefiltered through a 47 mm 8 µM Cyclopore track-etched membrane (Whatman, Milwaukee, USA) and a 0.45 µm Sterivex (Millipore-Sigam, Burlington, USA) sequentially. Samples were treated with 0.1% chloroform and left overnight at 4 °C. The next day, samples were precipitated with 10% Polyethylene Glycol 8000 (PEG, Fisher Scientific) and incubated at 4 °C overnight. These PEG viromes were then centrifuged at 14,000 × g for 2 h. The viral pellets were stored at −20 °C for DNA extraction. To extract the viral DNA, the Invitrogen™ Purelink™ Viral DNA kit (ThermoFisher, Carlsbad, CA) was used according to the manufacturer’s instructions. DNA concentrations were quantified using a Qubit 2.0 Fluorometer using a High-Sensitivity dsDNA kit (Thermo Fisher Scientific, Waltham, USA). Hereafter, these samples are referred to as seawater viral fraction.

### Sequencing, quality control, and assembly of metagenomic data

DNA library preparation and sequencing were performed by AZENTA, Inc. in South Plainfield, NJ, USA. The NEB NextUltra DNA Library Preparation kit was utilized according to the manufacturer’s instructions (NEB, Ipswich, MA, USA). The genomic DNA underwent fragmentation using acoustic shearing via a Covaris S220. The fragmented DNA was then subjected to end-repair and subsequent adapter ligation following the adenylation of 3′ ends. Adapter-ligated DNA underwent indexing and enrichment through limited cycle PCR. The resulting libraries were verified using TapeStation (Agilent Technologies, Palo Alto, CA, USA) and quantified using Qubit 2.0 Fluorometer and real-time PCR (Applied Biosystems, Carlsbad, CA, USA). Sequencing was performed on an Illumina HiSeq 4000 (Illumina, San Diego, CA, USA) in a 2 × 150 paired-end configuration. The base calling was performed using the HiSeq 4000 or equivalent Control Software, and the.bcl files were converted to fastq and demultiplexed using bcl2fastq v. 2.17.

All metagenomic reads were filtered and trimmed using BBduk, BBTools v.38.86 [79]. Read trimming used left and right sides (qtrim = rl, trimq = 30), adapter trimming of both ends with a k-mer size of 23 (ktrim = rl, k = 23, mink = 11), a hamming distance of one, and tpe/tbo parameters. Trimmed reads with a quality score of less than 30, entropy below 0.90, and sequence similarity to phiX were removed. The overall quality of the reads was assessed with FastQC v.0.11.9 [80]; all seawater reads and sargassum forward reads were of high quality. However, sargassum reverse reads contained a frontal G repeat region of 15 base pairs. This repeat region was removed using FastP v0.23.2 [81]. Illumina sequencing, followed by quality filtering, generated 9.3 × 10^7^ reads from sargassum, 8.2 × 10^7^ reads from the seawater cellular fraction, and 8.4 × 10^7^ from the seawater viral fraction. Quality-controlled reads for the sargassum and seawater cellular fractions were co-assembled independently using MEGAHIT v1.2.9 [82] using the meta-large flag and the minimum contig length of 1000 base pairs. The seawater viral fraction samples were assembled separately using SPAdes v.3.15.4 [83] with the metadata and minimum contig length of 1000 bp flags. Co-assemblies of the sargassum and seawater cellular fraction reads produced 2.4 × 10^4^ and 1.2 × 10^4^ contigs, respectively. Per sample assembly of the seawater viral fraction reads resulted in 4.8 × 10^5^ contigs.

### Identification of sargassum morphotype

To determine the morphotype of sargassum sampled, we used an alignment to complete mitochondrial genomes [57] and partial mitochondrial 16S genes [8] for *S*. *natans* I, *S*. *natans* VIII, and *S*. *fluitans* III obtained from GenBank. A BLASTn database was generated for all mitochondrial genomes and partial 16S genes, which were then aligned to the co-assembled sargassum contigs from this study. The results led to the conclusion that we sampled *S*. *natans* VIII, which is the morphotype name referred to when we discuss the metagenomic data.

### Taxonomic profiles of *S. natans* VIII and seawater bacterial reads

For both *S*. *natans* VIII and seawater cellular fractions, quality-controlled reads were taxonomically classified by Kaiju v.1.9.0 [84] with the proGenomes database [85] (accessed November 2022), which consists of a representative set of bacteria from proGenomes and viruses from the NCBI RefSeq. The mode used for this analysis was greedy, allowing for three mismatches, with a default minimum required match length and match score of 11 and 65, respectively. Unclassified reads were not included when calculating percent read abundances belonging to different bacterial and viral groups. 31.90% of *S*. *natans* VIII reads and 76.70% percent of seawater reads were classified as bacterial by Kaiju, with 66.84% of the *S*. *natans* VIII reads being unclassifiable using the proGenomes and viruses from NCBI RefSeq, which is expected because they do not include eukaryotic organisms. Bacterial community diversity, richness, and evenness were calculated using the percent read abundances from Kaiju at the species level.

### Generation of bacterial metagenome-assembled genomes

Bacterial metagenome-assembled genomes (bMAGs) were constructed for both *S*. *natans* VIII and seawater cellular fractions by mapping quality-filtered reads to MEGAHIT contigs using Bowtie2 v2.3.5 [86]. Using SAMtools v1.9 [87], the SAM files were compressed, sorted, and indexed according to binning algorithm specifications. Three binning programs were employed to construct bMAGs: CONCOCT v1.0 [88], MetaBAT2 v2.12.1 [89], and MaxBin2 v2.2.6 [90]. All three binners combine contig coverage and sequence composition to cluster contigs into genomes. The following raw bins were refined, re-assembled, taxonomically classified, and quantified using the *bin_refinement*, *reassemble_bin*, *bin_classifcation*, and *quant_bins* modules of MetaWRAP v1.3.2 [91]. Both refinement and reassembly modules use CheckM-genome v1.0.12 [92] to assess the completeness and contamination of all bins. For classification, MetaWRAP uses Taxtor-tk v1.3.3 [93] in conjunction with the NCBI nt and NCBI-tax databases (accessed November 2022). To obtain abundances for each bin, MetaWRAP calculates genomes per million reads by using a modified version of SALMON [94], which was initially adapted to calculate transcripts per million reads from RNA-seq data. bMAG abundance was estimated by calculating a length-weighted bMAG coverage of individual contigs relative to reads mapped to all co-assembled contigs (not all reads in the metagenome), allowing comparisons between samples with uneven numbers of eukaryotic reads. For further analysis, only bins with greater than 50% completion and less than 10% contamination were used. Note that while the 50% completion threshold prevents a complete assessment of the functional potential of the bin, which was not the objective of this study, it allows screening for viral sequences. *S*. *natans* VIII and seawater bMAGs were dereplicated separately at the 95% nucleotide identity within each sample set using the *anvi-dereplicate-genomes* module of Anvi’o v.7.1 [95] with fastANI v1.32 [96]. To generate the bMAG tree based on nucleotide identity, the bMAGs from both samples were combined and dereplicated at 85% nucleotide identity using the same procedures as above and visualized using the *infer* and *convert_to_itol* modules from GTDB-tk v2.1.1 [97].

### Identification of viruses and generation of viral metagenome-assembled genomes

MEGAHIT contigs for the *S*. *natans* VIII and seawater cellular fractions, SPades contigs for the seawater viral fractions, and bMAGs were separately dereplicated at 98% identity using CD-HIT v4.8.1 [98] and used to identify viral contigs with VIBRANT v1.2.1 [99]. Briefly, VIBRANT identifies open reading frames with Prodigal v2.6.3 [100], uses a Hidden Markov Model (HMM) to annotate viral proteins using three separated databases, KEGG, VOG, and Pfam, and employs a neural network based on genomic content to identify putative viral genome sequences. Viruses identified in bMAGs were screened for flanking bacterial sequences using VIBRANT and CheckV v1.0.1 [101] to identify confirmed proviruses. All viral contigs were binned to generate viral metagenome-assembled genomes (vMAGs) with vRhyme v1.1.0 [102], which groups viral contigs based on sequence composition and read coverage abundance. To dereplicate contigs within the vMAGs, BLASTn v2.12.0+ was used to identify repeat contigs, categorized as contigs with at least 97% identity and 95% coverage; repeat contigs were removed with Seqkit v2.2.0 [103]. To accommodate for vMAGs composed of several contigs, the contigs were N-linked with 200 N spacers using the vRhyme script *link*_*bin*_*sequences.py* [102]. All vMAGs and unbinned viral contigs of medium/high quality, or over 10 kb were combined and dereplicated using virathon (GitHub—Felipehcoutinho/Virathon: Genomic Analysis of Viruses of Archaea and Bacteria). Briefly, virathon creates viral populations using Prodigal v2.6.3 [100] to identify open reading frames, count the number of genes per sequence, and utilize an all-vs.-all BLASTn at the gene level to calculate the percentage of shared genes and ANI. Viral genomes with 80% shared genes and 95% ANI are considered to be within the same population, and a greedy approach is employed to retain the longest genome as the population representative. The resulting viruses comprise the high-quality *S*. *natans* VIII and seawater viral database (SSVdb) used for the remainder of the metagenomic analysis. VIBRANT also annotated temperate phage proteins, such as integrases, to identify putative temperate viruses. We added to this annotation by manually curating a database of integrases, excisionases, transposases, and recombinases from NCBI Viral RefSeq protein, pVOG, and Swissprot for a total of 2916 temperate phage-related proteins (accessed June 2023). Viruses were labeled as putative temperate based on BLASTp matches with an *e* value less than 10^−5^ between the SSVdb proteins and this database. The viruses in the SSVdb were taxonomically classified using Kaiju v.1.9.0 [84] with the viruses database, which consists of viruses from RefSeq (accessed August 2023). The mode used for this analysis was greedy, allowing for three mismatches, with a default minimum required match length and match score of 11 and 65, respectively.

### Viral fractional abundance

Reads from *S*. *natans* VIII*,* seawater cellular fraction, and seawater viral fraction were mapped to the SSVdb at 95% identity with SMALT v0.7.6 [104]. Fractional abundances (*f*(*i*)) were calculated using the equation below [105].

1 $$f\left(i\right)= \frac{r(i)}{T(j)} . \frac{L(\text{mean})}{L(i)}$$

where (*r*(*i*)) is the number of reads mapped to each contig obtained with SAMtools v1.9 [87], *L*(mean) corresponds to the mean viral genome length(bp), *L*(*i*) denotes the length of the viral genome, and (*T*(*j*)) represents the number of viral mapped reads in each metagenome/virome sample. If less than ten reads were mapped to a virus in a given sample, the virus was considered absent (fractional abundance = 0).

For the O-antigen biosynthesis and biofilm formation genes in the SSVdb heatmap (Fig. 4a), the median of the viruses was calculated per sample type. Every virus that encoded a gene was summed to get the fractional abundances per sample type. In the last fractional abundance-based figure, the AMG pathways encoded by the SSVdb (Sup. Fig. 3), the fractional abundance for each virus that encoded a gene for a specified pathway was summed to get the sum of the fractional abundance per sample. Viral fractional abundances were used to calculate diversity, richness, and evenness indexes.

### Viral proteomic tree

The viral proteomic tree was constructed based on an all-versus-all protein search between the SSVdb and reference viruses in the Genomic Lineages of Uncultured Viruses of Archaea and Bacteria (GL-UVAB) database (accessed on 04-06-2022) and International Committee on Taxonomy of Viruses (ICTV) database (accessed 11-03-2022) using *GLUVAB_v0.6.pl* [106]. GL-UVAB labels viruses as closest relatives with a minimum of 50% average amino acid identity (AAI) and 70% matched protein-encoding genes. Dice distances between all viruses were used to compute a neighbor-joining tree. To accommodate for N-linked vMAGs, the original script was edited with the prodigal -m flag, allowing for gene calling across N-links. The output newick file was imported into the Interactive Tree of Life v1.8 (iTol) for visualization and annotation.

### Viral genome annotations

In addition to the VIBRANT annotation with KEGG, Pfam, and VOG, structural annotation was supplemented from Phage Artificial Neural Network (PhANNs) for select viruses of interest. KOs for genes in the KEGG pathways “O-antigen nucleotide sugar biosynthesis”, “O-antigen repeat unit biosynthesis”, “Biofilm formation in *E*. *coli*”, “Biofilm formation in *P*. *aeruginosa*”, and “Biofilm formation in *V*. *cholera*” were identified in the viral database and used for analyses of biofilm-related genes.

### Phage-host pairs

The prokaryotic virus-host predictor tool (PHP) [107] was used to assign hosts at the order level using all bMAGs greater than 20% complete and less than 10% contaminated for the phages represented in the genome plots (except prophages) and the most abundant phages in each sample type. PHP employs a Gaussian model that uses differences in k-mer frequencies generated between viral and bacterial genomes to predict the infection relationship.

### Sargassum microbial community biofilm and induction assays

Sargassum samples, identified using the guidelines from Parr 1939, were collected nearshore (approximately 2 m from shore) before beaching close to the University of Miami’s Rosenstiel School of Marine, Atmospheric, and Earth Science (RSMAES) campus on May 16th, 2023. Samples were collected in autoclaved glass containers, placed in a cooler, and immediately returned to the main campus of the University of Miami for processing. One hundred fifty grams of sargassum were rinsed with DI water to remove seawater and loosely associated bacteria and transferred to a new autoclaved glass container. The organisms tightly associated with sargassum tissue were detached with a cell scraper and resuspended in 200 mL of 100 kDa-filtered seawater. After scraping, the algal tissue was transferred to six 250 mL centrifuge tubes with 200 mL of 100 kDa filtered seawater and vortexed for 20 min to remove the remainder of the detached community. This sargassum microbiome suspension was then filtered through a 100 µm filter mesh (AquaticExperts, USA) that was cleaned with a 10% bleach solution and rinsed thoroughly with DI water. The 10 µm-filtered sargassum microbiome suspension was centrifuged at 8000 × g for 30 min to pellet the microbial cells. The supernatant containing most of the extracellular viruses was discarded, and the pellets were washed a second time by resuspending in 100 mL of 100 kDa-filtered seawater and centrifuging again at 8000 × g for 30 min. The supernatant was discarded, and the pellets were resuspended, combined in 75 mL of 100 kDa filtered seawater and 25% glycerol, and stored at –80 °C for future use. This community is referred to here as the sargassum bacterial enrichment. Then, 50 µL of the sargassum bacterial enrichment was diluted into 950 µL of 100 kDa filtered seawater, fixed with 2% PFA, stained with 1 µL of 10X of SYBR Gold Nucleic Acid Gel Stain (Invitrogen, USA), filtered onto 0.02 µm Whatman Anodiscs (Cytiva, USA), and dried for 20 min in the dark. The Anodiscs were placed onto glass microscope slides with 15 µL of mounting solution (0.02 µm filtered 1X PBS, 1% ascorbic acid, and 50% glycerol) and a glass coverslip with an additional 15 µL of mounting solution. Slides were visualized under oil immersion at 63× objective using a ZEISS Axio Imager.A2 equipped with an Axiocam 506 mono camera and the X-Cite Mini (Excelitas Technologies, USA) and stored at −2 0 °C. Ten images were captured using the Zeiss software Zen. Cells were size delimited using Zen and counted manually to obtain bacterial cells per mL of sample.

### Biofilm assay with seawater viral community

To determine the effect that pelagic sargassum supplementation and viruses have on bacterial biofilm formation, six different 6-well plates (CytoOne, USA Scientific) communities were set up: one containing sargassum bacteria only grown in 10% sargassum-supplemented seawater, one sargassum bacteria and seawater viruses grown in sargassum-supplemented seawater, one containing sargassum bacteria grown in seawater, one containing sargassum bacteria and seawater viruses grown in seawater, one containing only seawater viruses in sargassum-supplemented seawater, and one containing only seawater viruses in seawater. Each plate consisted of five replicates and one blank for quantification. To make the 10% sargassum-supplemented seawater, 100 g of mixed sargassum was ground with a mortar and pestle, blended with 1 L of 0.45 µm seawater, filtered through a 100 µm mesh and 0.45 µm Sterivex filter, and autoclaved. Each well received: 5 mL of 100 kDa-filtered seawater or 5 mL of 0.45 µm-filtered autoclaved 10% sargassum-supplemented seawater. The wells that contained sargassum bacterial enrichment were inoculated with 7.1 × 10^6^ cells. The wells that contained seawater viruses had 7.1 × 10^7^ viral-like particles (VLPs) for a final MOI of 10 if the well contained bacteria and viruses. After the different communities were created, the plates were left to sit at 25 °C for 3 days. After 3 days, the supernatant from each well was removed and frozen at –80 °C. The wells were washed twice with 2 mL of 100 kDa filtered seawater and stained with 2 mL of 0.001% crystal violet (Eastman Organic Chemical, Rochester, USA) for 10 min. The crystal violet was removed, and each well was washed with 2 mL of 100 kDa filtered seawater four times. The plates were photographed, and 2 mL of 100% ethanol was added to put the crystal violet into solution. The OD540 for each plate was measured using a Synergy H1MD Hybrid Reader (BioTek, USA).

Plate 1 contained seawater, bacteria, and no viruses. Plate 2 had seawater, bacteria, and viruses. Plate 3 contained sargassum-supplemented seawater, bacteria, and no viruses. Plate 4 had sargassum-supplemented seawater, bacteria, and viruses. Plate 5 had seawater, no bacteria, and viruses. Plate 6 contained seawater, sargassum-supplemented seawater, no bacteria, and viruses.

### Planktonic induction assay

To investigate if prophages present within the sargassum bacterial community are inducible, we conducted an induction assay with the sargassum bacterial enrichment and two separate inducers, UV light and mitomycin C. Ten percent sargassum-supplemented seawater (5 mL at 10% final concentration) and sargassum bacterial enrichment (at 1.4 × 10^6^ cells/mL final concentration) were added to five replicate wells on three separate 6-well plates (CytoOne, USA Scientific). One well was not inoculated as a negative control. The communities were allowed to grow at room temperature (maintained at 25 °C) for 15 h and shaken at 180 rpm. The OD600 was recorded every 3 h except for an overnight period. One plate was induced with UV light (254 nm, 120 V, 60 Hz, 0.4 A, Spectro-UV) for 30 s, the second was induced with 1 µg/mL of mitomycin C, and the final plate received no treatment. The communities were left to grow for an additional 33 h with OD600 recording every 3 h, and 50 µL aliquots were taken pre-induction (t = 15 h) and 12 h post-induction (t = 27) for epifluorescence microscopy. These samples were prepared and imaged as stated above.

### Biofilm induction assay

To determine the impact that prophage induction has on biofilm formation, we conducted a biofilm induction assay with the sargassum bacterial enrichment and one inducing agent, mitomycin C. Sargassum-supplemented seawater (5 mL at 10% final concentration) and sargassum bacterial enrichment (at 1.4 × 10^6^ cells/mL final concentration) were added to five replicate wells on six separate 6-well plates (CytoOne, USA Scientific). Each group, mitomycin C treatment and control, had three plates assembled for biofilm quantification and microscopy at three time points: 12 h after inoculation (pre-induction), 12 h post-induction, and 24 h post-induction. After inoculation, the plates were left to grow at room temperature (maintained at 25 °C) with no shaking for 12 h. After 12 h, the media was removed from the first set of plates, and the wells were rinsed twice with 1 mL of 0.02 µm-filtered artificial seawater. The time frame for this experiment was adopted from previous works on biofilm assays with roseobacter species [56]. The biofilms were stained with 2 mL 0.01% crystal violet (Eastman Organic Chemical, Rochester, USA) for 10 min and subsequentially washed with 2 mL of 0.02 µm-filtered artificial seawater three times. For quantification, 2 mL of 100% ethanol was added to each well, and the OD540 was measured using a Synergy H1MD Hybrid Reader (BioTek, USA). At this time, the media was changed for the post-induction samples, and they continued to grow for another 12 and 24 h. The last two groups were quantified using the protocol above 12 h and 24 h post-induction. Five replicates of each treatment and control pre- and post-induction were scraped from the plates, and their DNA was extracted using the same DNA extraction protocol described above. Total DNA was sequenced on an Illumina HiSeq and the same bioinformatics pipeline for viral genome identification was applied.

### Statistics and figures

All statistics were performed in R. For the sargassum bacterial community growth curves (Fig. 5a), the bacterial cells/mL and VLPs/mL (Fig. 5b), the VMR (Fig. 5c), the OD540 measurements (Fig. 6a and b), and the biofilm induction experiment phage fractional abundances (Fig. 6d) data were first determined to be normal with the Shapiro Wilks test using dplyr v2.3.2, if the data were not normally distributed a Wilcoxon rank-sum was used with the stats v4.2.2 package. Equal variance was then determined with a Levene’s test using car v3.1–2. If variances were equal, a Two-sample *T* test or an ANOVA was carried out with the stats v4.2.2 package. If variances were unequal, a Welch’s *T* test was substituted in the same package. Following ANOVAs, a Tukey HSD test was used to determine the detailed differences within the data. All boxplots show the median and the interquartile range, which is composed of the 25^th^ and 75^th^ percentiles. All heatmaps were created with R-package pheatmap v.1.0.12. The percent read abundance heatmap (Fig. 1b) was normalized by taking the log of the percent read abundance. Both the viral fractional abundance (Fig. 3b), O-antigen biosynthesis/biofilm formation gene (Fig. 4a), and metabolic gene (Sup. Fig. 3) heatmaps were normalized by column using the scale function in pheatmap. All bar plots, boxplots, and line graphs were plotted using R-packages ggplot2 v3.4.1 and ggExtra v0.10.0. The viral genome maps were constructed using the R-package genoPlotR v0.8.1. The bMAG percent nucleotide identity tree (Fig. 2a) was constructed using GTDB-tk v2.1.1, and the viral proteomic tree (Fig. 3a) was created using GL-UVAB. The newick file for both trees was visualized and annotated using iTol v1.8.

### Supplementary Information

Supplementary Material 1: Supplementary Fig. 1. Bacterial order abundances. Mean abundances of *S. natans* VIII and seawater bacteria at the order level calculated from reads annotated by Kaiju. Supplementary Fig. 2. *S. natans* VIII and seawater bMAG completeness. Boxplot comparison of bMAG completeness for both sample types (Two-sample t-test, *p*-value = 0.343). Supplementary Fig. 3. Auxiliary metabolic gene pathway comparison between *S. natans* VIII, seawater cellular, and seawater viral communities. Fractional abundances indicate the sum of all viruses encoding a gene in the specified pathway. The heatmap was normalized by pathway using a z-score function. Supplementary Fig. 4. Sargassum bacterial enrichment. Representative epifluorescence microscopy image showing sargassum bacterial enrichment depleted of extracellular viruses. Bacteria and viruses were stained with SYBR Gold and visualized at 6300X magnification. Supplementary Fig. 5. Mitomycin C and control groups during biofilm induction assay. Representative epifluorescence microscopy images of mitomycin C-treated (bottom) and untreated (top) sargassum bacterial enrichment communities 12 h post-induction. Bacteria and viruses were stained with SYBR Gold and visualized at 6300X magnitude. Supplementary Fig. 6. *Vibrio* spp. abundances (log-transformed percentages) in *S. natans* VIII and seawater cellular fractions. Median abundance of *Vibrio* sp. of log percent read abundance per sample type (*S. natans* VIII *n* = 5, Seawater *n* = 5). Supplementary Table 1. Bacterial community ecology statistics. Shannon diversity index, Simpson diversity index, richness, and evenness for both bacterial communities. Supplementary Table 2. Number of viruses identified per sample and their predicted infection strategies. Supplementary Table 3. Viral community ecology statistics. Shannon diversity index, Simpson diversity index, richness, and evenness. Supplementary Table 4. Viral host prediction for viruses of interest discussed in the text.

Supplementary Material 2: Supplementary Data 1. Sequencing and quality control data for 2021 metagenome samples.

Supplementary Material 3: Supplementary Data 2. Taxonomic annotation of 2021 sargassum samples based on mitochondrial genome and 16S gene BLASTn comparisons with databases.

Supplementary Material 4: Supplementary Data 3. Bacterial abundances and mapped read counts at the phylum level.

Supplementary Material 5: Supplementary Data 4. Bacterial abundances and mapped read counts at the order level.

Supplementary Material 6: Supplementary Data 5. Bacterial MAG quality, taxonomy, abundance, and prophage presence.

Supplementary Material 7: Supplementary Data 6. Fractional abundances, genome length, and infection strategy for all viruses in the SSVdb.

Supplementary Material 8: Supplementary Data 7. Viral-encoded metabolic genes in the SSVdb grouped by KEGG pathway indicated by the KO number.

Supplementary Material 9: Supplementary Data 8. Viral-encoded biofilm genes in the SSVdb and corresponding KEGG pathway indicated by the KO number.

## Data Availability

Raw reads are available in the NCBI Sequence Read Archive (PRJNA1063307 for metagenomes of naturally occurring S. natans VIII (Figs. 1, 2, 3 and 4) and PRJNA1111456 for metagenomes from biofilm induction assay (Fig. 6)). Contigs [108], S. natans VIII bMAGs [109], seawater bMAGs [110], and the SSVdb [111] are available in Figshare under the project Sargassum Viruses and Bacteria (https://figshare.com/projects/Sargassum_viruses_and_bacteria/191235). The code used for the bioinformatic pipeline is available at https://github.com/Silveira-Lab/Stiffler_etal_2024/blob/main/Stiffler_etal_2024.sh.
