# Supplementary material for: Genomic and induction evidence for bacteriophage contributions to sargassum-bacteria symbioses
Source: Microbiome. 2024 Aug 1;12:143. doi: 10.1186/s40168-024-01860-7 (PMC11295528; doi:10.1186/s40168-024-01860-7)
Supplement: Supplementary file 2 — Supplementary Material 1: Supplementary Fig. 1. Bacterial order abundances. Mean abundances of S. natans VIII and seawater bacteria at the order level calculated from reads annotated by Kaiju. Supplementary Fig. 2. S. natans VIII and seawater bMAG completeness. Boxplot comparison of bMAG completeness for both sample types (Two-sample t-test, p-value = 0.343). Supplementary Fig. 3. Auxiliary metabolic gene pathway comparison between S. natans VIII, seawater cellular, and seawater viral communities. Fractional abundances indicate the sum of all viruses encoding a gene in the specified pathway. The heatmap was normalized by pathway using a z-score function. Supplementary Fig. 4. Sargassum bacterial enrichment. Representative epifluorescence microscopy image showing sargassum bacterial enrichment depleted of extracellular viruses. Bacteria and viruses were stained with SYBR Gold and visualized at 6300X magnification. Supplementary Fig. 5. Mitomycin C and control groups during biofilm induction assay. Representative epifluorescence microscopy images of mitomycin C-treated (bottom) and untreated (top) sargassum bacterial enrichment communities 12 h post-induction. Bacteria and viruses were stained with SYBR Gold and visualized at 6300X magnitude. Supplementary Fig. 6. Vibrio spp. abundances (log-transformed percentages) in S. natans VIII and seawater cellular fractions. Median abundance of Vibrio sp. of log percent read abundance per sample type (S. natans VIII n = 5, Seawater n = 5). Supplementary Table 1. Bacterial community ecology statistics. Shannon diversity index, Simpson diversity index, richness, and evenness for both bacterial communities. Supplementary Table 2. Number of viruses identified per sample and their predicted infection strategies. Supplementary Table 3. Viral community ecology statistics. Shannon diversity index, Simpson diversity index, richness, and evenness. Supplementary Table 4. Viral host prediction for viruses of interest discussed in the text [file 40168_2024_1860_MOESM1_ESM.pdf]

Supplementary Figures and Tables

Genomic and induction evidence for bacteriophage contributions to sargassum-bacteria symbioses

Alexandra K. Stiffler[1], Poppy Hesketh-Best[2], Natascha S. Varona[1], Ashley Zagame[1], Bailey A. Wallace[1], Brian LaPointe[3], Cynthia Silveira[1,4*]

Supplementary Figures

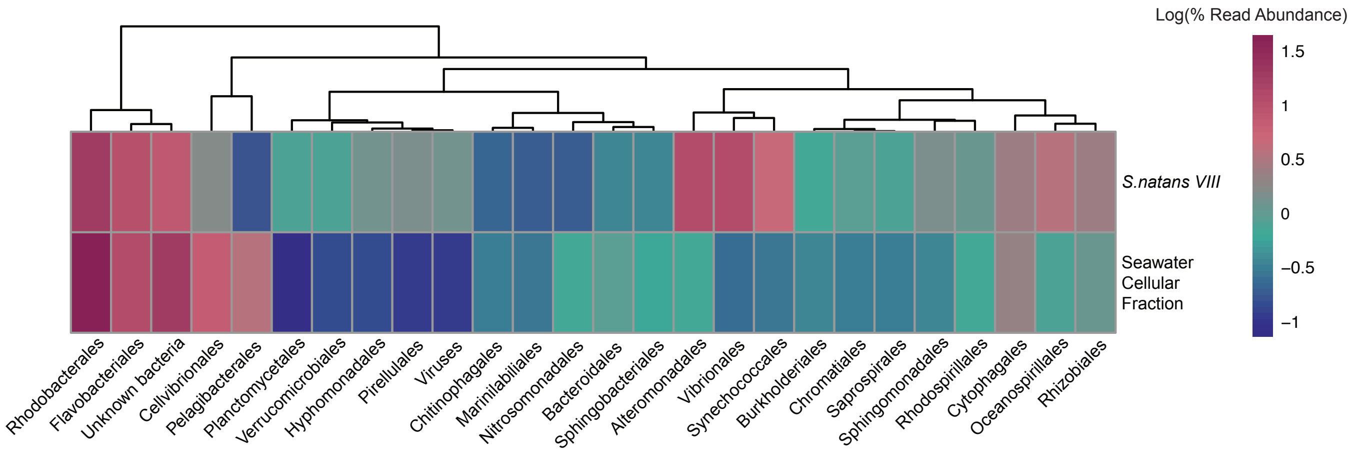

Supplementary Fig. 1 | Bacterial order abundances. Mean abundances of *S. natans* VIII and seawater bacteria at the order level calculated from reads annotated by Kaiju.

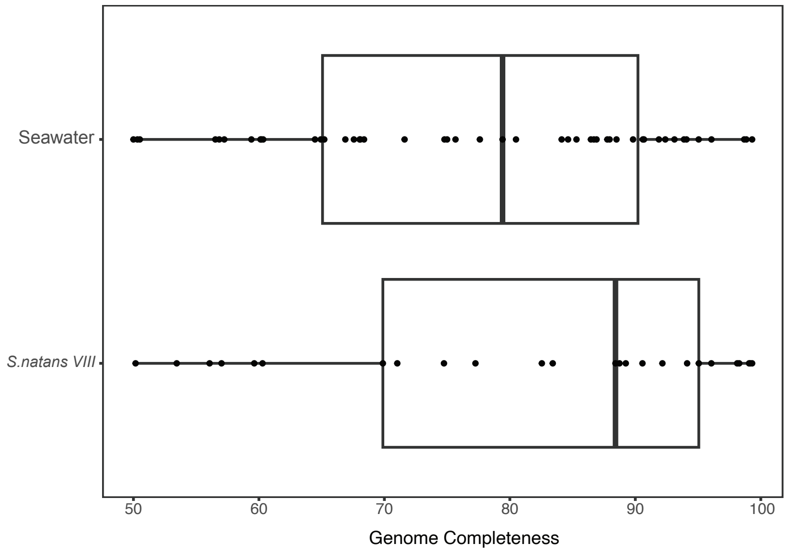

Supplementary Fig. 2 | *S. natans* VIII and seawater bMAG completeness. Boxplot comparison of bMAG completeness for both sample types (Two-sample t-test, p-value = 0.343).

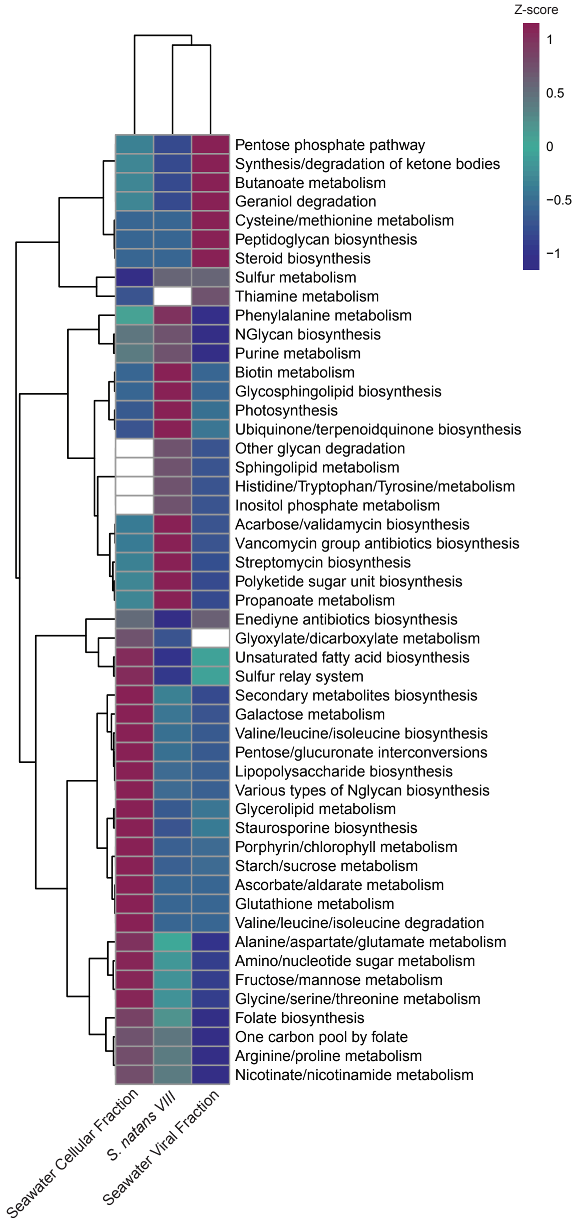

Supplementary Fig. 3 | Auxiliary metabolic gene pathway comparison between *S. natans* VIII, seawater cellular, and seawater viral communities. Fractional abundances indicate the sum of all viruses encoding a gene in the specified pathway. The heatmap was normalized by pathway using a z-score function.

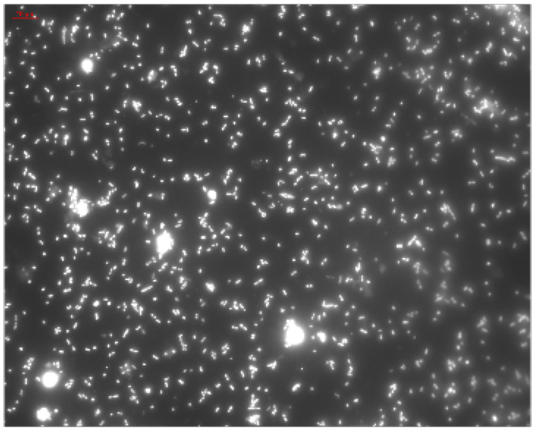

Supplementary Fig. 4 | Sargassum bacterial enrichment. Representative epifluorescence microscopy image showing sargassum bacterial enrichment depleted of extracellular viruses. Bacteria and viruses were stained with SYBR Gold and visualized at 6300X magnification.

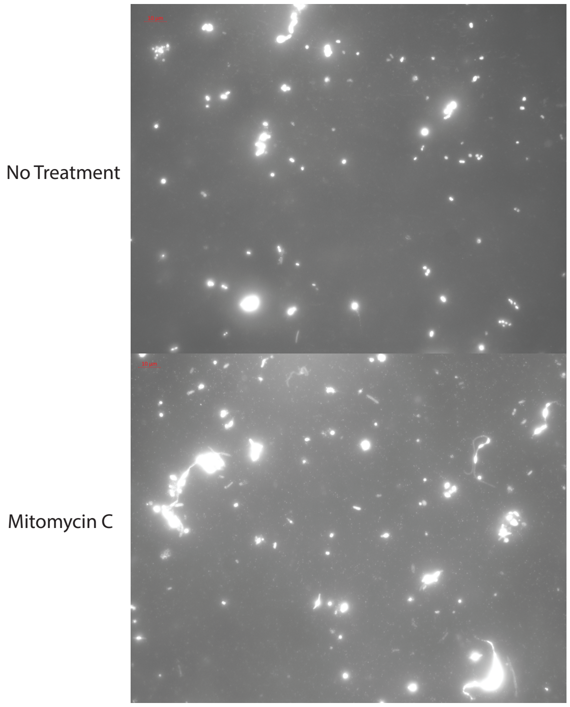

Supplementary Fig. 5 | Mitomycin C and control groups during biofilm induction assay. Representative epifluorescence microscopy images of Mitomycin c-treated (bottom) and untreated (top) sargassum bacterial enrichment communities 12 hours post-induction. Bacteria and viruses were stained with SYBR Gold and visualized at 6300X magnitude.

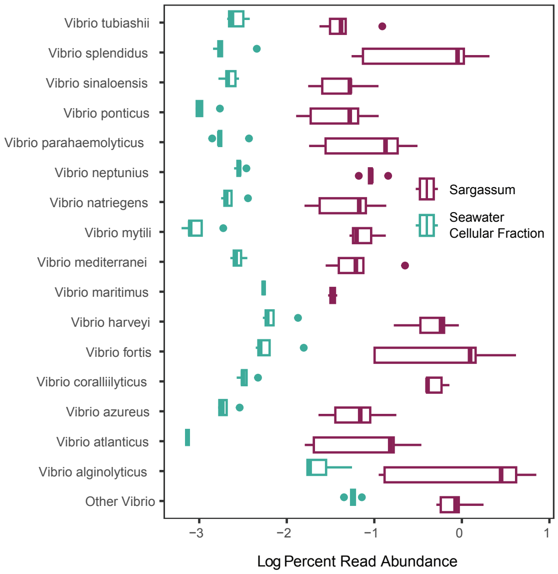

Supplementary Fig. 6 | *Vibrio* spp. abundances (log-transformed percentages) in *S. natans* VIII and seawater cellular fractions. Median abundance of *Vibrio* sp. of log percent read abundance per sample type (*S. natans* VIII *n* = *5*, Seawater n = 5).

Supplementary Tables

Supplementary Table 1 | Bacterial community ecology statistics. Shannon diversity index, Simpson diversity index, richness, and evenness.

	Shannon Index	Simpson Index	Richness	Evenness
S. natans VIII	5.852	0.943	10871	0.630
Seawater cellular	3.896	0.848	10677	0.420

Supplementary Table 2 | Number of viruses identified per sample and their predicted infection strategies.

**Sample**	**# Unique Viruses**	**# Lytic**	**# Temperate**	**Lytic: temperate ratio**
S. natans VIII 1	1557	1109	448	2.475
S. natans VIII 2	2388	1753	635	2.761
S. natans VIII 3	2438	1810	628	2.882
S. natans VIII 4	1951	1378	573	2.405
S. natans VIII 5	2546	1886	660	2.858
Seawater cellular 1	1709	1259	450	2.798
Seawater cellular 2	1790	1328	462	2.874
Seawater cellular 3	1937	1437	500	2.874
Seawater cellular 4	2051	1537	514	2.99
Seawater cellular 5	2044	1525	519	2.938
Seawater viral 1	3616	2768	848	3.264

Seawater viral 2	3609	2765	844	3.276
Seawater viral 3	3593	2757	836	3.298
Seawater viral 4	3565	2739	826	3.316
Seawater viral 5	3571	2742	829	3.308

Supplementary Table 3 | Viral community ecology statistics. Shannon diversity index, Simpson diversity index, richness, and evenness.

	Shannon Index	Simpson Index	Richness	Evenness
S. natans VIII	5.682	0.985	2176	0.741
Seawater cellular	5.825	0.991	1906	0.772
Seawater viral	7.464	0.999	3591	0.912

Supplementary Table 4 | Viral host prediction for viruses of interest discussed in the text.

**Phage Genome**	**PHP Max Score**	**Host Bin**	**Host Taxonomy**	**Reason for discussion**
Sarg_vRhyme_bin_429	1441.505	Sargassum_bin.41	Pseudoalteromonas	most abundant phage in Sargassum
Sarg_vRhyme_bin_16	1409.422	Sargassum_bin.16	Flavobacteriaceae	second most abundant phage in Sargassum
sarg_contig.k127_118014	1447.564	Seawater-Sterivex_bin.63	Alphaproteobacteria	third most abundant phage in Sargassum
SW-St_vRhyme_bin_206	1426.899	Sargassum_bin.74	Flavobacteriaceae	most abundant phage in Seawater cellular fraction
SW-vir_vRhyme_bin_4054	1430.496	Seawater-Sterivex_bin.28	Bacteria	second most abundant phage in Seawater cellular fraction

SW-vir_vRhyme_bin_4055	1380.585	Seawater-Sterivex_bin.28	Bacteria	third most abundant phage in Seawater cellular fraction
sw_sterivex_contig.k127_823716	1452.096	Sargassum_bin.68	Rhodobacterales	most abundant phage in Seawater viral fraction
SW-vir_vRhyme_bin_2182	1451.202	Seawater-Sterivex_bin.112	Proteobacteria	second most abundant phage in Seawater viral fraction
sw-virome-2.NODE_107	1312.229	Sargassum_bin.94	Alteromonas	third most abundant phage in Seawater viral fraction
Sargassum_bacterial_bin_72_1	Prophage	Sargassum_bin.72	Gammaproteobacteria	First biofilm phage genome plot
Sarg_vRhyme_bin_472	1435.621	Seawater-Sterivex_bin.117	Flavobacteriaceae	Second biofilm phage
Sarg_vRhyme_bin_31	1442.201	Seawater-Sterivex_bin.117	Flavobacteriaceae	Third biofilm phage genome plot
Sargassum_bacterial_bin_92_7	1391.363	Sargassum_bin.92	Rhodobacterales	Fourth biofilm phage genome plot
